# The Hebrew Physicians of Mediæval Avignon

**Published:** 1910-10-08

**Authors:** 


### SPECIAL ARTICLE.

### THE HEBREW PHYSICIANS OF MEDIÆVAL AVIGNON.

An article by Dr. P. Pansier, of Avignon, in *Janus* gives an account of his interesting researches with regard to the Jewish medical practitioners who flourished in that town from the thirteenth to the fifteenth centuries. The importance of the Jewish colony of Avignon may be gauged from the fact that the Emperor Frederick II., in 1178, put it under the protection of the bishop to guard it from the rapacity of the counts and nobles. At first inhabiting a quarter outside the ramparts, the colony in 1221 moved into the town, settling in the parish of St. Pierre, to the prior of which each family paid a due of *un denier raymondin*. The records of the thirteenth century give little information of the state of medicine in Avignon at the time beyond an account of the conditions upon which a licence was granted to practise as a physician or as a barber-surgeon, and a requisition to the *speciatores*, or drug-sellers, forbidding them to enter into any understanding with the physicians as to commissions on the medicines ordered by the latter. There would seem to have been no civil law prohibiting a Jew practising medicine, in spite of the fact that in 1241 the Council of Albi, presided over by Zeon, Bishop of Avignon, declared that all Christians, who in case of illness had recourse for treatment to a Jewish physician, should be excommunicated, an edict often ignored by rich and poor alike. Moreover, the Faculty of Medicine of Montpellier appears to have granted its diplomas to Hebrew practitioners. There is even reason to suppose that the promulgation of such an edict by the Council was largely influenced by a desire to prevent the flooding of the "Midi" of France with Jewish physicians. For many years a flourishing school of medicine had existed at Lunel, near Montpellier, presided over, among others, by a Spanish Jew, Judas ben-Tibbon, who subsequently taught medicine at the Faculty of Montpellier, as also did his son Salomon after him. The author has succeeded in tracing in the public records of Avignon the names of fifteen practitioners of medicine and surgery in the thirteenth century, one of whom, Vitalis, was practising there in 1282, and is mentioned as "medicus Judeus," but he has found no trace of any public edict ratifying the veto pronounced by the Council of 1241.

### The Avignon Edict.

The following century saw a great increase in the prestige of Jewish physicians, which led to the following edict of the Council of Avignon in 1337: "Among Christians, to the shame of the Catholic religion, a pernicious custom has prevailed. The very persons who ought to despise the vile services of Jews as coming from an enemy, rush to them when they assume some vague title of surgeon or physician, in order to discover, as they imagine, some relief to, but more accurately some aggravation of their pains." It was, therefore, enjoined that only in case of the gravest emergency, when no Christian help was at hand, was it allowable to have recourse to a Jewish practitioner. In 1306 Robert, eldest son of Charles II. of Provence, had published a similar edict, but this had not been enforced in Avignon, and, in fact, had been constantly evaded in Provence. In 1341 a Synod, held at Avignon, removed the restriction pronounced in 1337, as it was found that many Christians ran the risk of going without medical help at all in times of sickness, owing to the rapidly diminishing numbers of Christian physicians. Such excommunications as had already been pronounced were, moreover, cancelled. The Synodial statutes, published in 1366 by Aubert Arnault, Archbishop of Auch, and administrator of the vacant Bishopric of Avignon, contained no provisions against Jewish practitioners, though forbidding the employment of Jewish midwives and the suckling of Christian children by Jewish nurses and *vice versa*.

### The Popularity of Jew Doctors.

It may be interesting to trace the causes of the great respect in which Hebrew physicians were held. Their popularity undoubtedly rested on their greater knowledge of therapeutic measures. The training of the orthodox practitioner was mainly theoretical, consisting in the reading of a few books of Galen, fragments of the canons of Avicennus, the treatise of Johanitius on the pulse, and the treatise of Theophilus on the urine. If the candidate for a diploma could dissert for a given time on a phrase taken at random from any of these authors, he was allowed to practise, though entirely ignorant of patients and the art of looking after them. His subsequent results in private can be imagined. The requirements for a surgeon were an apprenticeship to a master of the art, a vague knowledge of a few fragments of Roger, Lanfranc, or later Guy de Chauliac, and a purely empirical education in the use of the lancet, plasters, bandages, the setting of fractures, and the reduction of dislocations. Among the Jews, however, the art of healing was usually transmitted from father to son, though in some cases a boy was apprenticed to a co-religionist. Instruction, both theoretical and practical, was given, the student taking part in the daily routine of the master's practice. The Jews were thus better clinicians, and secured better results than their Christian rivals. Though for the most part physicians, they practised medicine and surgery indifferently, and had none of the pride which prevented the Christian practitioner from performing the most ignoble details of medicine and surgery. Most of the Jewish physicians added the profession of money-lending to their other accomplishments, with the result that they became rich more quickly than their better-paid Christian *confrères*. They would, nevertheless, seem to have been met in consultation by the latter on friendly and courteous terms.

The Emancipation of the Jew Doctor.

The fifteenth century saw a law passed by which the Jews had liberty to practise medicine and surgery in Avignon, the municipal statutes even containing a clause exempting Jewish practitioners from the usage whereby Jews were obliged to remain in their own quarter of the town from the Wednesday in Holy Week until after the ringing of the bells on Holy Saturday. Records exist which show that many convents were attended by Jewish practitioners. In 1374 the convent of the Cordeliers had Abraham de Carcassone as physician, paying him a salary of four florins a year. In 1420 the same convent paid two florins to Durandus Tamani, "medico nostro." The advent of Albericus Andree to the town, at the request of the Consuls, to teach and practise medicine, resulted in the Jewish being supplanted by the Christian practitioner that same year. On the death of the latter, in 1431, however, Tamini was reinstated, and in 1459 he was replaced by Salomon Ferussol. Thus three out of four of the physicians attached to this convent were Jews. The public authorities during the same period had no hesitation in taking advantage of the services of Hebrew practitioners during periods of stress caused by pestilence and war, some of them even being attached to the hospitals. The names of sixty Jewish physicians have been traced as practising at Avignon during the fifteenth century, a considerable number in view of the diminution of the population consequent upon the departure of the Papal Court. Most of them, in addition to their own profession, followed that of money-lending or entered into partnership with their wives in commercial undertakings. In the sixteenth century the number of Jewish physicians diminished considerably. In 1555 they were prohibited from practising in Avignon and the county of Venaissin. In the seventeenth century no Jewish physicians existed, as orthodoxy was required before a candidate could be admitted to a degree. The Jewish physician thus disappears or becomes an irregular practitioner—a bonesetter or sorcerer.

